# Extracellular Vesicular miRNA in Pancreatic Cancer: From Lab to Therapy

**DOI:** 10.3390/cancers16122179

**Published:** 2024-06-08

**Authors:** Prashant Kumar Tiwari, Poojhaa Shanmugam, Vamika Karn, Saurabh Gupta, Richa Mishra, Sarvesh Rustagi, Mandeep Chouhan, Devvret Verma, Niraj Kumar Jha, Sanjay Kumar

**Affiliations:** 1 Biological and Bio-Computational Lab, Department of Life Science, School of Basic Sciences and Research, Sharda University, Greater Noida 201310, Uttar Pradesh, India; 2 Amity Institute of Biotechnology, Amity University, Mumbai 410206, Maharashtra, India; 3 Department of Biotechnology, GLA University, Mathura 281406, Uttar Pradesh, India; 4 Department of Computer Engineering, Parul University, Ta. Waghodia, Vadodara 391760, Gujarat, India; 5 School of Applied and Life science, Uttaranchal University, Dehradun 248007, Uttarakhand, India; 6 Department of Biotechnology, Graphic Era (Deemed to be University), Dehradun 248002, Uttarakhand, India; 7 Centre for Global Health Research, Saveetha Medical College, Saveetha Institute of Medical and Technical Sciences, Saveetha University, Chennai 602105, Tamil Nadu, India; 8 School of Bioengineering & Biosciences, Lovely Professional University, Phagwara 144411, Punjab, India; 9 Department of Biotechnology, Sharda School of Engineering and Technology, Sharda University, Greater Noida 201310, Uttar Pradesh, India

**Keywords:** extracellular vesicle, pathogenesis, metastasis, drug resistance, pancreatic cancer, diagnostic biomarkers

## Abstract

**Simple Summary:**

The elusive nature of pancreatic cancer frequently results in late diagnoses, which in turn leads to unfavorable treatment outcomes. The existing screening methods lack reliability, and conventional treatments demonstrate limited efficacy. Recent studies have revealed that pancreatic cancer cells interact with their surroundings, specifically through small particles called extracellular vesicles (EVs). These EVs, secreted from cells, carry important molecules, such as miRNAs, proteins, mRNAs, and lipids, that influence cancer growth, metastasis, and response to treatment. Understanding this intercellular communication among cells and microenvironment may help to find a new potential target for novel therapeutic strategies.

**Abstract:**

Pancreatic cancer is a prevalent lethal gastrointestinal cancer that generally does not show any symptoms until it reaches advanced stages, resulting in a high mortality rate. People at high risk, such as those with a family history or chronic pancreatitis, do not have a universally accepted screening protocol. Chemotherapy and radiotherapy demonstrate limited effectiveness in the management of pancreatic cancer, emphasizing the urgent need for innovative therapeutic strategies. Recent studies indicated that the complex interaction among pancreatic cancer cells within the dynamic microenvironment, comprising the extracellular matrix, cancer-associated cells, and diverse immune cells, intricately regulates the biological characteristics of the disease. Additionally, mounting evidence suggests that EVs play a crucial role as mediators in intercellular communication by the transportation of different biomolecules, such as miRNA, proteins, DNA, mRNA, and lipids, between heterogeneous cell subpopulations. This communication mediated by EVs significantly impacts multiple aspects of pancreatic cancer pathogenesis, including proliferation, angiogenesis, metastasis, and resistance to therapy. In this review, we delve into the pivotal role of EV-associated miRNAs in the progression, metastasis, and development of drug resistance in pancreatic cancer as well as their therapeutic potential as biomarkers and drug-delivery mechanisms for the management of pancreatic cancer.

## 1. Introduction

Globally, pancreatic cancer ranks seventh in terms of mortality. However, in the Europe and U.S., it ranks fourth after lung, colorectal, and breast cancer. Furthermore, it has been predicted that pancreatic cancer is expected to reach the third position of mortality by 2030 [1]. The pancreas is a complex organ with dual functions that act as a bridge between the digestive and endocrine systems. Patients suffering from pancreatic cancer do not have any obvious symptoms in the beginning, and this is the reason it is not diagnosed in the initial stages. The study has revealed that pancreatic cancer progression is associated with non-aggressive tumors that spread within the pancreatic ducts, which is specifically called pancreatic intraepithelial neoplasia [2,3]. Although there are several types of pancreatic cancer, pancreatic ductal adenocarcinoma (PDAC) is recognized as the most common type, which approximately accounts for 90% of all pancreatic malignancies, and it develops from cells lining pancreatic ducts [4]. Due to the lack of advanced medical tools, substantial difficulties in early detection of PDAC are being observed, which causes delayed diagnosis of patients at advanced stages, making surgery difficult in cases of locally advanced or distant metastases [5]. Several studies have suggested that the tumor microenvironment significantly influences the initiation and progression of PDAC [6].

Extracellular vesicles (EVs) are a heterogeneous group of lipid bilayer membrane-bound structures released from various cells, including pathogenic and non-pathogenic cells. They are composed of a range of bioactive molecules, including a variety of proteins, lipids, and genetic material such as DNA and diverse types of RNAs (mRNA and microRNAs), which play a crucial role in significant intercellular interactions for maintaining the physiology of the cells [6]. EV-associated micro-RNAs (EV-miRNAs), have been implicated in various types of cancer, including pancreatic cancer, in terms of their role in cancer progression as well as their therapeutic potential [7]. miRNAs are diminutive, non-coding RNA molecules that regulate gene expression post-transcriptionally by binding to messenger RNAs (mRNAs). In cancer, dysregulation of miRNAs is frequently observed, contributing to disease progression [8]. They influence tumor behavior by targeting various proteins and immune checkpoints, affecting tumor proliferation, differentiation, and development [9]. Encapsulation of miRNAs within exosomes protects them from enzymatic degradation, allowing stable transport through the extracellular environment. This unique mode of miRNA delivery has important implications for cell-to-cell communication and offers potential therapeutic applications, particularly in cancer [10]. This review aimed to provide a comprehensive overview of the various types of EV-miRNAs that play diverse roles in pancreatic cancer progression, metastasis, and development of drug resistance, as well as their therapeutic potential as a biomarker for early diagnosis of pancreatic cancer.

## 2. Extracellular Vesicle (EVs)

Secretion of extracellular vesicles is an evolutionarily conserved process present in all kingdoms of life [11]. In the field of fundamental biology, research on EVs focuses on understanding the biogenesis and release of these inherent carriers as well as their subsequent behavior when interacting with target cells. This includes an investigation of the genotypic and phenotypic responses generated by EVs as well as the mechanisms through which they facilitate cell-to-cell communication [12].

Based on size and biogenesis, EVs have been generally categorized into three categories: (a) Exosomes range in size from 30 to 150 nanometers (nm), making them the smallest among cellular vesicles, and are synthesized through endocytic pathways in the cells; (b) Microvesicles are 40–1000 nm in size, which are formed through budding from plasma membranes; and (c) Apoptotic bodies represent some of the largest EVs, having a size greater than 1000 nm and synthesized during the apoptosis. EVs have been isolated from a variety of bodily fluids, including plasma, amniotic fluid, saliva, urine, breast milk, semen, and body fluids [13,14,15,16]. Exosomes contain specific biomarkers such as CD81, CD63, CD9, HSP70, MHC-I, TSG101, and LAMP1, distinguishing them from other EVs. These markers are crucial for identifying and studying exosomes in various biological contexts. In contrast, microvesicles are characterized by the presence of biomarkers like CD40, CD62, and integrin. The unique profiles of these biomarkers in exosomes and microvesicles facilitate their respective roles in intercellular communication, diagnostics, and therapeutic applications [16,17].

### Biogenesis of Exosomes

Exosome biogenesis initiates from early endosomes inside the cells, then after post-maturation, they transform into late endosomes. During this transformation, specific cargoes such as proteins, coding and non-coding RNAs, DNA, and lipid molecules capture and form multivesicular bodies (MVB) and undergo inward budding, leading to the formation of intraluminal vesicles (ILVs) which detach from the membrane. These ILVs are released outside by exocytosis. This process is primarily conducted by two pathways: the endosomal sorting complex required for transport (ESCRT) dependent pathway and the ESCRT independent pathway [18].

**Figure 1:**
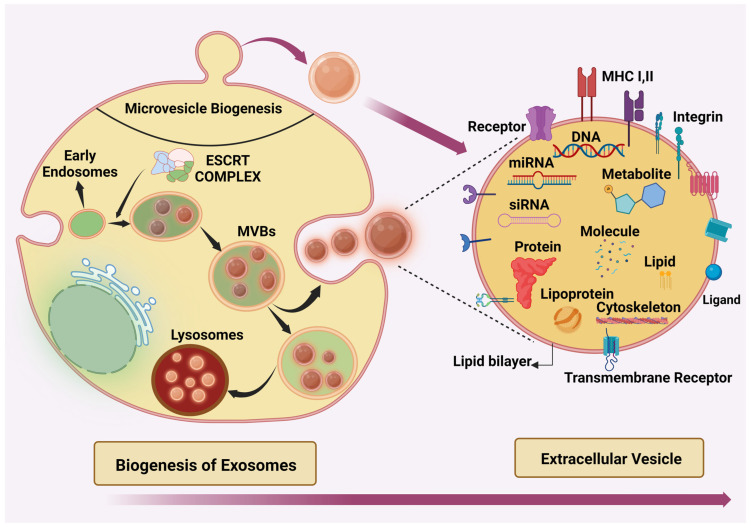
Exosomes arise from intraluminal vesicles (ILVs) within multivesicular bodies (MVBs), a process facilitated by the ESCRT complex or alternative ESCRT-independent pathway. Upon ILV formation, MVBs undergo trafficking toward either the plasma membrane or the lysosome. At the plasma membrane, fusion of MVBs is mediated by the SNARE complex, culminating in exosome release. On the contrary, microvesicles emerge by budding directly from the plasma membrane. Apoptotic bodies, exclusive to apoptotic cells, are shed from the cell surface. These extracellular vesicles are lipid bilayer-enclosed and contain various biomolecules including nucleic acids (DNA and RNA), diverse proteins, receptors, lipids, and metabolites.

The ESCRT complex system consists of approximately 30 proteins, organized into four complexes: ESCRT-0, ESCRT-I, ESCRT-II, and ESCRT-III [19]. Initiation of ILV formation occurs concurrently with the loading of cargo on the membrane of MVBs, and this is mediated by the multivalent ubiquitin-binding ESCRT-0 complex. This group includes hepatocyte growth factor-regulated tyrosine kinase substrate (HRS) and signal transducing adaptor molecule (STAM) [20]. ESCRT-0 has ten ubiquitin-binding sites, facilitating the capture of polyubiquitylated cargo. Upon binding of the ubiquitinated cargo to the ESCRT-0 complex, the HRS-STAM complex initiates the recruitment of ESCRT-1, which, aided by the TSG-101 protein, transports the cargo. Concurrently, ESCRT-1 recruits ESCRT-II by interacting with its VPS28 and VPS36 subunits. Subsequently, ESCRT-II facilitates the recruitment of ESCRT-III. ESCRT-III is critically involved in the formation of MVBs, a specialized structure involved in cargo sorting for degradation. ESCRT-III recruits charged MVB protein 2-4 (CHMP2-4) polymers, crucial for assembling the ESCRT-III complex [18,19]. In addition, the accessory protein AAA-ATPase VPS4 functions to dismantle and recycle ESCRT-III [21] (Figure 1).

## 3. Application of EV-miRNAs in Pancreatic Cancer

miRNAs are short non-coding RNA molecules ranging from 19 to 24 nucleotides in length [22]. Their primary functions have been shown in the silencing, degradation, and modification of mRNA post-transcriptionally [23]. To perform these functions, they utilize the base-pairing principle forming either complete or partial binding with the 3′ UTR regions of mRNA [24]. Several types of human cancers, including PDAC, have been associated with alterations in miRNA expression. In one study, PDAC patient plasma has shown around twenty circulatory miRNAs (CIR-miRNAs) at higher levels than in healthy individuals, suggesting their potential as diagnostic biomarkers for PDAC [25]. Regarding exosomes, some research suggests that during intercellular communication, they significantly influence the biological characteristics of cancer cells and enhance their invasiveness by promoting angiogenesis and immune evasion. Similarly, it was also observed that exosomes released by cancer-associated fibroblasts (CAFs) provide a variety of metabolites to cancer cells, and these active metabolites help cancer to spread effectively even in nutrient-deprived environments [26]. Exosomes obtained from PDAC can promote proliferation, migration, and invasive abilities in specific cell lines MiaPaCa-2 and AsPC-1 [27].

### 3.1. Role of Exosome-miRNAs in Progression of PDAC

Exosomes are enriched with diverse microRNAs and they have important effects on physiological and pathophysiological processes [28], just for this reason relationship between exosome-miRNA and tumors has always been of interest to researchers because this information is highly valuable in the field of medical science, like what functions do these miRNAs serve in the development and advancement of tumors, and how they are associated with angiogenesis, growth, migration, metastasis, invasion, immune escape, drug resistance, and apoptosis [29].

### 3.2. Role of Exosome-miRNAs in Migration, Invasion, and Metastasis in PDAC

Various EV-miRNAs, including miRNA-222, miRNA-501-3p, and miRNA-5703, have been documented to influence the progression of PDAC, particularly in the context of migration, invasion, and metastasis [30]. Similarly, a study conducted by Guangbing Xiong and colleagues found that the overexpression of miR-10a-5p is also associated with migration and invasion in AsPC-1 and T3M4 cell lines, Alternatively, the knockdown of miR-10a-5p in AsPC-1 and Su86.86 cells reduces cell migration. Importantly, miR-10a-5p had no significant effect on cell proliferation, apoptosis, or cell cycle [31]. The molecular mechanism underlying these observations revealed that miR-10a-5p promotes epithelial-mesenchymal transition (EMT) by up-regulating genes such as vimentin, Slug, and Snail [31], and up-regulation enhances the migratory and invasive abilities of PDAC cells. Additionally, miR-10a-5p can reduce the expression of E-cadherin and claudin-1 during this process [31]. In the study on the miRNA-21, it was noticed that the artificial introduction of this miRNA into cancer-associated fibroblasts (CAFs) increased the ability of PDAC cells by secretion of crucial substances, such as metalloproteases, cytokines, and growth factors, resulting in increased growth and metastasis [32]. In pancreatic cells (PC-1.0), it was recognized that overexpression of EVs-miR-125b-5p is associated with invasion and metastasis. For this purpose, it actively targets the MEK2/ERK2 cell signaling pathway. Additionally, it inhibits the tumor-suppressor STARD13 [33]. The JAK/STAT pathway is known for its involvement in immunological responses and tumor development. miRNA-301a regulates these pathways potentially and targets crucial component STAT3, which contributes to proliferation and metastasis [34].

In one study, researchers found that in PDAC, EV-miRNAs influence the expression of various genes responsible for tumor progression through different pathways. For example, tumor-derived exosomal-miRNA-222 increases p27 phosphorylation leading to an increased AKT signaling pathway, resulting in proliferation and invasion of tumor cells [35]. In a separate study, it was also noticed that exosomal-miRNA-501-3p from tumor-associated macrophages (TAMs) enhance tumorigenesis and metastasis by affecting the transforming growth factor-beta receptor 3 (TGFBR3) and modulating the TGF-beta signaling pathway [36]. Chemokine-like factor (CKLF) like MARVEL Transmembrane domain-containing protein 4 (CMTM4) is a tumor suppressor protein [37], and it reduces tumorigenesis in colorectal, renal, and brain tumors. Studies also revealed that overexpression of CMTM4 inhibits the growth of cancer cell lines [38].

**Figure 2:**
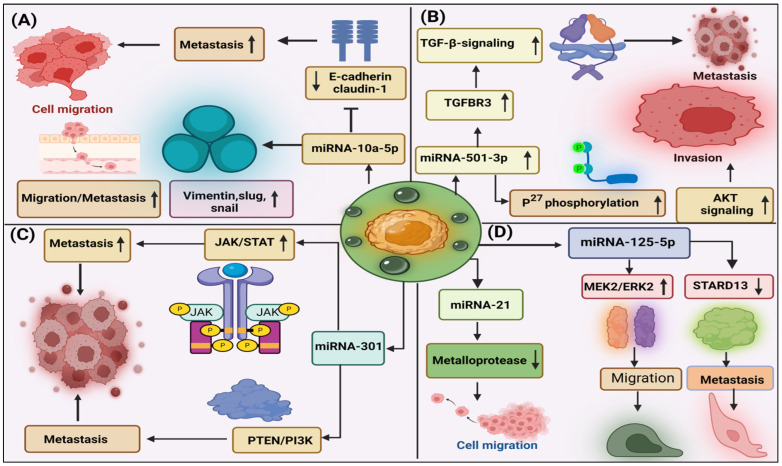
(**A**) Schematic representation depicting the miRNA-10a-5p induces the expression of transcriptional factors Vimentin, Slug, and Snail while suppressing E-cadherin and Claudin1, ultimately facilitating cell migration and metastasis. (**B**) miRNA-501-3p targets TGFBR3, leading to the activation of TGF-β signaling and phosphorylation of P27, thereby inducing AKT signaling and promoting invasion. (**C**) miRNA-301 in metastasis through JAK/STAT and PTEN/PI3K pathways. (**D**) miRNA-125-5p promotes migration and metastasis by inducing MEK2/ERK2 and inhibiting STARD13, whereas miRNA-21 induces metalloprotease activity, thereby promoting cell migration. (The direction of arrow shown in the box, ↑—increased expression; ↓—decreased expression; ⊣—inhibition).

Exosomal lncRNA and cirRNAs also play crucial roles in various cancers, lncRNAs SBF2 antisense RNA 1 (SBF2-AS1), has been shown to have higher expression in liver cancer (HULC). Other exosomal RNAs, such as NONHSAT105177, SOX2 overlapping transcript (Sox2ot), phosphodiesterase 8A (PDE8A), and isoleucyl-tRNA synthetase (IARS), have been associated with migration, invasion, and metastasis in PDAC [39]. TGF-β signaling pathways play an important role in PDAC, and two pathways associated with this are the canonical Smad pathway and the noncanonical non-Smad signaling pathway. In PDAC, TGF-β activates several non-Smad pathways like PI3K/AKT, JNK/p38, and ERK/MAPK. These pathways generally have a role in PDAC progression [40]. Under a hypoxic environment, PDAC secretes exosome-miRNA-301a with high levels, and it targets PTEN/PI3K signaling, resulting in the polarization of M2 macrophages and metastasis in PDAC [41] (Figure 2 and Table 1).

### 3.3. Role of Exosome-miRNAs in Proliferation and Angiogenesis of PDAC

Proliferation and angiogenesis play critical roles in PDAC development, and several results of various studies show that exosome-miRNA influences the level of various genes and also activates many signaling pathways, resulting in the advancement of cancer [35]. In the human genome, miRNA-222, (a member of the miRNA221/222 family), was located on the p11.3 region of the X chromosome. It has been shown that dysregulation of miRNA-22 has been associated with various types of cancer [47]. Additionally, miRNA-222 increases p27 phosphorylation by activating Akt signaling pathways resulting in PDAC proliferation [48]. Exosomal miRNA-5703 derived from pancreatic stellate cells (PSCs), down-regulates the activity of CMTM4 and increases the activity of the PI3K/Akt pathway; resulting in cell proliferation in pancreatic cancer cells [48]. The B-cell translocation gene (BTG) functions as a tumor suppressor gene pivotal in regulating cell-cycle progression, apoptosis, and cellular differentiation across different cancer types, including lymphoid and solid tumors. miRNA-27a down-regulates BTG gene activity, resulting in angiogenesis in pancreatic cancer [49].

The RAS/ERK signaling cascade is frequently associated with cancer cells. Mutations occurring in RAS proteins lead to their constitutive activation, thereby triggering signaling along the ERK pathway, resulting in uncontrolled cellular proliferation and increased survival [50]. Pancreatic stellate cells (PSCs) continue to secrete miRNA-21-enriched exosomes, which play a crucial role in activating the RAS/ERK pathway in cancer cells, leading to epithelial-to-mesenchymal transition (EMT), resulting in tumor growth [51].

### 3.4. Role of Exosome-miRNAs in Immune Escape in PDAC

**Figure 3:**
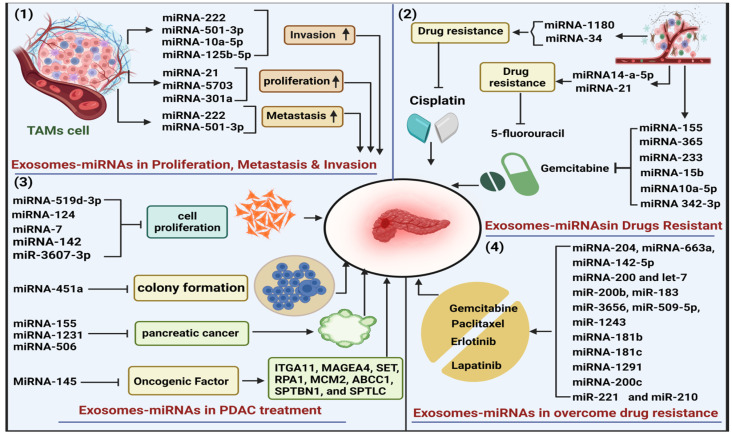
Exosome-associated miRNAs are involved in various roles such as (**1**) invasion, proliferation, and metastasis; (**2**) drug resistance; (**3**) PDAC treatment; and (**4**) overcoming drug resistance. The direction of arrow shown in the box, ↑—increased expression.

Immunity is regulated by a complex networking of various cells and molecules that protect the body from various infections, including cancers. Due to mutation in genes and uncontrolled growth of cancer cells, various types of antigens are produced. These antigens assist in identifying cancer cells by the different verity of the immune response and help in destroying them, thus helping to inhibit the progression of cancer [52]. EVs have been extensively studied for their involvement in immunity. Researchers propose various mechanisms explaining how exosomes may protect cancer cells from the immune response and contribute to cancer progression [53]. One study has demonstrated that EVs secreted from cancer cells facilitate immunological escape by suppressing immune-cell activation, resulting in functional impairment of the immune response [54]. Dendritic cells (DCs) are the primary antigen-presenting cells (APCs) crucial for initiating immune responses, which enhance the expression of Toll-like receptors and produce a variety of cytokines. TLR4 has been shown to exhibit significant anti-tumor activity [55]. In pancreatic cancer, exosomal miRNA-203 has been shown to down-regulate IL-12 as well as TLR4 in DCs, which may assist cancer cells in completing immune evasion [56]. Another study reported that exosomal miRNA-212-3p reduced the level of RFX-associated protein (RFXAP), thereby reducing the level of MHC II and initiating DC-induced immunological tolerances. This piece of evidence suggests that EV-miRNAs may have immunosuppressive properties, enabling pancreatic cancer cells to safely exit from immune reactions [57] (Figure 3).

### 3.5. Role of Exosome-miRNAs in PDAC as Diagnosis Biomarker

Pancreatic cancer poses a significant challenge in early detection, primarily due to its less obvious symptoms. The lack of timely diagnosis has led to a surge in cancer incidence and subsequently increased the mortality rate among patients [58]. Conventional imaging tests including ultrasound, CT scan, and MRI are commonly employed in the diagnostic assessment of pancreatic cancer [59]. Despite CA-199 being an FDA-approved biomarker for pancreatic cancer, its accuracy in detecting early-stage cancer is unsatisfactory due to its low sensitivity and its overall limited specificity [48]. Therefore, there is a need for an accurate, non-invasive technique to diagnose pancreatic cancer promptly. As previously discussed, exosomes, with their rich biological cargo, have emerged as promising candidates for the early detection of various cancers [60]. The findings of several studies have claimed that exosomal miRNA holds greater potential for diagnosis than peripheral blood-free miRNA. The main reason for this is that they are derived from original cells and contain a significant amount of RNAs, providing comprehensive insight into their source [61]. miRNA-driven transcriptional regulation has been shown to be involved in almost all cells. Additionally, altered expression of miRNAs has been related to various processes of pancreatic cancer, such as proliferation, epithelial-mesenchymal transition, metastasis, invasion, apoptotic escape, and chemoresistance [62]. Recent advancements in technology, including microarray, NGS, quantitative fluorescence probe-based PCR, in situ hybridization, and Northern blotting using locked nucleic acid (LNA) modified probes, have given several specific miRNAs, which could be employed in the diagnosis of various diseases [63]. Critically, increased miRNA21 expression emerges as a biomarker that not only differentiates patients with pancreatic cancer from healthy groups but also effectively delineates between various pancreatic conditions, including chronic pancreatitis and intraductal papillary mucinous neoplasm, etc. [45]. Similarly, miRNA-21, numerous other miRNAs overexpressed in cancers including miR-10a/b, miR-27a, miR-106a, miR-194, miR-196a/b, miR-210, miR-221/222, miR-301a-3p, miR-367, miR-375, miR-429, and miR-486 [42]. Furthermore, several miRNAs have been associated with higher chances of relapse cases and poor prognosis of pancreatic cancers. For instance, miRNA-451a has been correlated with higher chances of recurrence of pancreatic cancer [64]. Another study revealed a significant correlation between the overexpression of miR-222 and patients diagnosed with pancreatic cancer. This association suggests a possible link to larger tumor size and advanced TNM stages [35]. Bloomston and his team discovered that six miRNAs (miR-30a-3p, miR-105, miR-127, miR-187, miR-452, and miR-518a-2) indicate a better prognosis (survival of more than 2 years) in pancreatic cancer patients [65]. In one study, it was observed that miR-212 and miR-675, which are novel RNA molecules, and their overexpression are associated with the progression of different types of human cancers including pancreatic cancer. The down-regulation of tumor-suppressor miRNAs, such as Let-7g, miR-187, and miR-148a, was an independent predictor of a worse prognosis in pancreatic cancer patients [66]. In pancreatic cancer, it has been observed that the down-regulation of miR-142-5p and miR-204 is related to chemoresistance, whereas the overexpression of these miRNAs is associated with better overall survival in pancreatic cancer patients [67].

Certain miRNAs that influence specific signaling pathways involved in cancer development are concentrated in primary tumor exosomes, exceeding the levels that are found in normal cells [68]. It has been shown that EVs present in the blood plasma of patients with pancreatic cancer contained miRNA-451a, which was associated with the prognosis of the disease’s recurrence [69]. Additionally, a higher level of miRNA-23b-3p has been seen in EVs isolated from pancreatic cancer cells under in vitro conditions, which was associated with cell proliferation, migration, and invasion [6]. Furthermore, EV miRNA-339-5p has been demonstrated to be involved in the regulation of migration and invasion activities in pancreatic cancer cells [70].

Madhavan et al. found that the expression levels of miRNA-4306, miRNA-1246, miRNA-3976, and miRNA-4644 extracted from serum are associated with pancreatic cancer. Their research showed that these miRNAs were up-regulated by 83% in pancreatic cancer compared to non-pancreatic cancer patients [71]. Glypican-1 (GPC1) is a cell surface protein involved in cellular processes. In various cancers, including pancreatic cancer, GPC1 is studied as a potential biomarker [72]. Xianyin Lai compared EV-miRNA and GPC1 between PDAC, chronic pancreatitis (CP), and healthy controls. The results showed that GPC1 couldn’t diagnose PDAC as effectively as the increased expression of miR-10b, miR-21, miR-30c, and miR-181a. Additionally, it was found that low levels of miR-let7a could differentiate PDAC from controls and CP. After PDAC resection, elevated exosomal miRNA levels returned to normal within 24 h, with miR-10b and miR-30c significantly elevated in all PDAC cases [72]. Similarly, Nakamura et al., conducted a study utilizing pancreatic juice samples from 35 patients (27 patients with PDAC and 8 patients with chronic pancreatitis (CP)), and found that the expression of EV-miRNA-21 and EV-miRNA-155 were up-regulated in PDAC patients compared to CP patients. The study results concluded that EV-miRNA-21 and EV-miRNA-155 could differentiate between PDAC and CP patients with high accuracy (90% and 89%, respectively) [73]. Makoto Abue and his colleagues conducted a study on circulating miR-483-3p as a biomarker. They included 32 patients with PDAC, 12 patients with intraductal papillary mucinous neoplasm (IPMN), and 30 control patients. The results revealed that the expression level of miR-483-3p was significantly higher in comparison to HC and IPMN, respectively (*p* < 0.01, *p* < 0.05) [74] (Figure 3, Table 2).

### 3.6. The Role of Exosome-miRNAs in Drug Resistance of PDAC 

Gemcitabine is a type of chemotherapy drug commonly used to treat various cancers, including PDAC, by inhibiting DNA synthesis and inducing apoptosis [85]. miRNAs act as key regulators involved in cellular processes in a variety of cancers, including PDAC. Dysfunction of miRNAs leads to alterations in cellular pathogenesis. Interestingly, the dysregulation of miRNA expression contributes to drug resistance in PDAC [86]. Girijesh and colleagues found that in PDAC, miRNA-155 confers resistance to gemcitabine by up-regulating the ROS-detoxifying genes SOD2 and CAT and down-regulating the gemcitabine-metabolizing gene DCK [87]. Up-regulation of miRNA-17-5p reduces apoptosis and confers resistance to gemcitabine by reducing the expression of the BIM protein (Bcl-2 interacting mediator of cell death) [88]. In a study, it was observed that exogenous expression of miRNA-365 from TAM conferred resistance to gemcitabine in PDAC by down-regulating the apoptosis-promoting molecules SHC1 and BAX [89]. Numerous studies indicate that miRNAs have a very crucial function in regulating the EMT/MET process, which is pivotal in cancer cell invasion and drug resistance. For instance, miRNA-233 regulates the EMT process in PDAC cells by down-regulating FBW7 and up-regulating NOTCH-1, resulting in resistance to gemcitabine [90]. In an independent study, researchers observed that the up-regulation of miRNA-15b in drug-resistant PDAC (MiaPaCa-2) can promote EMT by targeting SMURF2 (HECT-family ubiquitin ligase (E3)) [91]. Similarly, another study revealed the involvement of miR-296-5p in regulating PDAC invasion and EMT characteristics by targeting Bcl-2 family proteins, resulting in PDAC drug resistance [92]. miRNA-301a is a hypoxia-sensitive miRNA in PDAC. It makes cancer cells drug resistant by targeting PTEN and P63 [74,93].

Furthermore, cancer-associated fibroblasts (CAFs) secrete exosomal miR-146a and miR-106b, which affect transcriptional pathways. Specifically, exosomal miR-106b has been shown to activate downstream signaling by targeting TP53INP1 [94]. Zhiyong Yang and his colleagues conducted a study on gemcitabine-resistant PDAC, revealing in their findings that exosomes originating from these cells have the ability to horizontally transfer the drug-resistance trait through miRNA-210 [88]. Over-expression of KLF6 in PDAC suppresses proliferation, metastasis, and EMT. Conversely, miR-342-3p inhibits the expression of KLF6, leading to resistance to gemcitabine in tumor cells [95]. In pancreas cells, transcription factor activating protein 2 gamma (TFAP2C) maintains cellular processes by inhibiting cell invasion and migration. Oncogenic miRNA-10a-5p induces drug resistance to gemcitabine by modulating TFAP2C [31]. Recent research has unveiled that circadian disruption is linked to the pathogenesis of tumors, whereas the circadian clock plays a crucial role in tumor suppression. PDAC secretes miRNA-35b-BMAL1-YY1, which is responsible for tumorigenesis and drug resistance. YY1 activates miR-135b level via interacting with its promoter, leading to the suppression of BMAL1, which is an important gene regulator of the circadian clock [96]. In pancreatic cancer, overexpression of miRNA-125a, miRNA-320c, and miRNA-1246 suppressed the activity of cyclin G2 (CCNG2), SMARCC1 (chromatin subfamily C member 1), and A20 (a zinc finger family protein), resulting in reduced drug sensitivity [97]

Cisplatin, also known as (SP-4-2)-diamminedichloridoplatinum (II), is a highly effective and widely used drug for many solid tumors including cervical, testicular, ovarian, and other cancers. It exhibits anticancer activity through multiple mechanisms, primarily by inducing DNA lesions through interactions with purine bases, followed by activation of various signaling pathways, ultimately leading to apoptosis [98]. Patients treated with Cisplatin chemotherapy have had initial success, but drug resistance limits its continued use. Various complex molecular mechanisms contribute to this resistance in cancer cells, including increased transporter expression, enhanced DNA repair, altered cell aggregation, drug inactivation, and facilitated drug efflux [99]. NF-κB, a vital protein complex, regulates cellular responses to stimuli like inflammation and cancer. TNIP2, a tumor suppressor, inhibits NF-κB signaling. Oncogenic miRNA-1180 boosts pancreatic cancer by regulating TNIP2 and causing Cisplatin resistance. Inhibiting miRNA-1180 enhances Cisplatin efficacy, inducing cancer cell apoptosis [100]. Schreiber et al. investigated miRNA involvement in Cisplatin resistance by comparing miRNA levels in Cisplatin-resistant pancreatic cancer cells (BxPC3-R) with parental cells (BxPC3) exposed to increasing Cisplatin concentrations. They found significant expression changes in 57 miRNAs, with 23 down-regulated and 34 up-regulated in the resistant cells. Employing an HMM algorithm, they pinpointed miR-374b down-regulation as potentially linked to drug resistance acquisition [101]. miRNA-34 regulates tumor-cell behavior by targeting Notch, c-Met, and Bcl-2 expression, which are associated with tumor-cell self-renewal and survival. Down-regulation of miRNA-34 affects p53-mediated apoptosis. Additionally, restoring miR-34a levels could boost the sensitivity of pancreatic cancer to the drug Cisplatin [102].

The antibiotic doxorubicin, obtained from the bacterium *Streptomyces pucetius* and synthesized in 1960, stands out as a potent chemotherapeutic agent within the anthracycline group. Its efficacy extends to various solid cancers in patients including ovarian, breast, bladder, and pancreatic cancers [103]. Doxorubicin interacts primarily with DNA base pairs, causing DNA strand breaks. Additionally, it affects the synthesis of both DNA and RNA by inhibiting the enzyme topoisomerase II, resulting in DNA damage and initiation of apoptosis [104]. Doxorubicin resistance is mainly associated with the induction of autophagy; suppression of autophagy processes effectively reduces or increases resistance to doxorubicin in various cancer groups. Emerging data indicated that specific miRNAs are very crucial in the development of resistant to doxorubicin drugs in pancreatic cancer [105]. Additionally, miR-142 plays a role in sensitizing pancreatic cancer cells to doxorubicin by suppressing DJ-1 and subsequently activating PTEN, inactivating the PI3K/AKT signaling pathway, which is often correlated with cancer survival and drug resistance [106].

5-fluorouracil (5-FU) is an anti-metabolite drug that has been widely used since 1957 in various types of cancer such as breast cancer, colorectal cancer, pancreatic cancer, etc. for cytotoxicity; it interferes with the biosynthetic activity by inhibiting thymidylate synthase (TS). Despite the many advantages of 5-fluorouracil, its diminished effectiveness is attributed to the development of resistance in cancer cells [107]. Pancreatic cancer cells respond to 5-fluorouracil in different ways and the mechanisms of resistance in cancer cells are not yet fully understood. Some studies have shown that several tumor-suppressive and carcinogenic miRNAs are associated with resistance to 5-fluorouracil [108]. In pancreatic cancer, a large proportion of oncogenic miRNAs, particularly miRNA-221, miRNA-320, and miRNA-21, play an important role in conferring resistance to 5-fluorouracil. Specifically, overexpression of miRNA-21 targets PTEN and PDCD4 through the PI3K/AKT/mTOR pathways. As a result, this activation promotes tumor proliferation and promotes resistance to 5-fluorouracil within cancer cells [109]. The Hippo signaling pathway plays a crucial role in organ size, regeneration, and hemostasis. Dysregulation of the Hippo pathway has been associated with a variety of cancers, including pancreatic cancer. Overexpression of miRNA-181c increases drug resistance to 5-fluorouracil in cancer cells by inhibiting the Hippo signaling pathway [96,97]. In an in vitro study, it was revealed that elevated expression of miRNA-14a-5p regulates carcinogenesis. Furthermore, it was observed that this increased expression leads to resistance to 5-fluorouracil by inhibiting tumor necrosis factor receptor (TNFR)-associated factor 6 (TRAF6) (Figure 3) [110].

### 3.7. Role of Exosome-miRNAs in Overcome Drug Resistance

Apart from promoting pancreatic cancer, many miRNAs help in overcoming drug resistance and tumor suppression. Overexpression of some miRNAs, such as miRNA-204, miRNA-663a, and miRNA-142-5p, is associated with decreased resistance to drugs such as gemcitabine [111]. Zinc finger E-box binding homeobox 1 (ZEB1), a transcription factor, plays an important role in drug resistance by inducing the EMT process, leading to tumor invasion and metastasis. Up-regulation of miRNA-200 and let-7 inhibits ZEB1, thereby preventing EMT and cell invasion, resulting in increasing sensitivity to gemcitabine [112]. In another study, it was observed that the expression of miR-200b and miR-183 was down in gemcitabine-resistant cells. Kruppel-like factor 4 (KLF4), an evolutionarily conserved transcription factor protein that plays an important role in cellular processes, directly increases the expression of miR-200b and miR-183 while inhibiting the expression of ZEB1, thereby increasing sensitivity to gemcitabine [31,96,113]. Similarly, miR-1243 miR-3656, and miR-509-5p have the ability to improve the sensitivity of pancreatic cancer cells to gemcitabine (GEM) by regulating processes associated with EMT [114] (Table 3 and Figure 3).

In pancreatic cancer, the higher level of BCL2 has been associated with the involvement of miRNA-21, which consequently enhances anti-apoptotic effects. BCL2 is targeted by miRNA-181b, leading to a decrease in its expression. Additionally, transfection of miRNA-181b also diminishes gemcitabine resistance [121]. FOLFIRINOX is a chemotherapy regimen used to treat PDAC. It comprises four medications: 5-fluorouracil (5-FU), leucovorin, irinotecan, and oxaliplatin [122]. It was widely adopted after the PRODIGE 4/ACCORD 11 trials, which showed significantly longer survival in patients receiving FOLFIRINOX compared to patients receiving gemcitabine after pancreatic surgery. Some studies suggest that miRNAs may predict drug resistance/sensitivity during FOLFIRINOX treatment [123]. In a clinical trial, it was found that when nanoparticle albumin-bound paclitaxel (Nab-paclitaxel) and gemcitabine were administered together in patients with advanced pancreatic cancer, an improvement in survival was observed within two months, without drug toxicity [124]. However, overexpression of miRNA-181c increases resistance to paclitaxel [118]. Furthermore, researchers noted that miRNA-1291 increases sensitivity to gemcitabine–nab-paclitaxel combination therapies via induced DNA damage and causing mitotic arrest [119]. Overexpression of miRNA-200c confers sensitivity to erlotinib drugs in PDAC. Mechanistically, it binds to the 3′ UTR region of Mig-6 mRNA, thereby reducing its level. The correlation between the Mig-6 (mRNA)/miR200c ratio and erlotinib sensitivity was significantly observed in both cancer cell lines and primary human tumor xenografts in vivo [119]. A potential strategy for treating PDAC involves the dual inhibition of human epidermal growth factor receptor 2 and epidermal growth factor receptor. Lapatinib, functioning as a competitive dual tyrosine kinase inhibitor, effectively interacts with both HER2 and EGFR. The highly expressed miR-221 and miR-210 have been linked to resistance against Lapatinib. Results from in vitro studies suggest that inhibition of miR-221 and miR-210 can enhance sensitivity to Lapatinib in PDAC [120].

### 3.8. Role of Exosome-miRNAs in PDAC Treatment

In an in vivo experiment, Hongwei Sun and colleagues found that NK cells have the ability to inhibit the malignant transformation of PC cells by affecting their proliferation, migration, and invasion. This inhibitory effect was determined to be mediated by miR-3607-3p, which is enriched in EVs derived from NK cells and transferred to PC cells. Further investigation revealed that miR-3607-3p exerts its effects by directly targeting interleukin-26 (IL-26) in PC cells [125]. Song Shang studied EV-derived miRNA-1231 and found that low expression of miRNA-1231 is significantly associated with the TNM stage of pancreatic cancer and can promote proliferation and migration. It was also observed that when miRNA-1231 derived from bone marrow mesenchymal stem cells (BM-MSCs) is externally administered to pancreatic cancer cells (BxPC-3 and PANC-1); it negatively regulates various cellular processes. This suggests that exosome-carried miR-1231 may suppress the aggressiveness of pancreatic cancer cells [30]. It has been demonstrated that miRNA-145 could suppress the level of insulin-like growth factor 1 receptor (IGF1R) and target pluripotency maintenance factors such as OCT4, SOX2, NANOG, and KLF4 [126]. The exogenous miR-145 in pancreatic cancer cells results in decreased expression of various cancer-associated genes important for pancreas carcinogenesis, such as SPTLC1, MCM2, SET, RPA1, ABCC1, MAGEA4, SPTBN1, and ITGA11 [127]. In a study, it was noted that miRNA-21 expression is associated with pancreatic intraepithelial neoplasia (PanIN) in a K-ras mutant mouse model. Increased expression of miRNA-21 in patients reduces anti-proliferative activity and anti-apoptotic effects. In contrast to this, the low expression of miRNA-21 is associated with adjuvant therapy. Hence, anti-miRNA-21 approaches can increase the activity of anti-cancer drugs [127]. The Mut L homolog 1 (MLH1) gene is recognized as the mismatch repair (MMR) gene. Mechanistic studies demonstrate that MLH1 expression is diminished in pancreatic cancer, and transfection of miRNA-155 can suppress MLH1 expression. The knockdown of miRNA-155 results in decreased cell growth and reduction in the expression of EGFR, MT1-MMP, and K-ras [13].

Tumor suppressor miRNA-451a inhibits proliferation and colony formation by regulating the expression of ATF2, YWHAZ, and RAB14. However, it has also been observed that its down-regulation is correlated with metastasis and low progression in gastric cancer [128]. Additionally, another miRNA-506 is up-regulated in pancreatic cancer, which is linked with reduced disease progression [129]. Under hypoxic conditions, overexpression of hypoxia-inducible factor-1α (HIF-1α) occurs alongside a notable decrease in miRNA-141 expression, facilitating cancer invasion. However, increased miRNA-142 expression has been shown to target HIF-1α, leading to inhibition and consequently reduced invasion and proliferation. Moreover, the miR-142/HIF-1α axis regulates key markers of epithelial-mesenchymal transition (EMT), including E-cadherin, VEGF-C, and Vimentin, which play crucial roles in cancer metastasis and progression [130]. In a study, it was observed that the expression level of miRNA-519d-3p is low in pancreatic cancer samples and cell lines, while an increased expression level was noticed for ribosomal p1rotein small subunit 15A (RPS15A). Additionally, RPS15A has been shown to significantly regulate β-catenin expression and the Wnt signaling pathway, both of which are implicated in cancer prognosis. The up-regulation of miRNA-519d-3p targets RPS15A, resulting in reduced activity of the Wnt/β–catenin cascade, ultimately leading to the inhibition of proliferation in these cells [131]. miRNA-124 expression levels are significantly low in pancreatic cancer. Elevated levels of miRNA-124 counteract the activity of the oncogene Rac1, which plays a key role in the MKK4-JNK-C-JUN pathway associated with cancer progression. Through negative regulation, miRNA-124 reduces the expression of Rac1, resulting in the inhibition of cell proliferation [132].

Furthermore, EV-miRNA derived from pancreatic cancer has the capability to target pivotal mutations, thereby contributing to the therapeutic management of pancreatic cancer. KRAS, TP53, SMAD4, and CDKN2A are four major driver genes for pancreatic cancer [3]. Mutant forms of the GTPase KRAS cause pancreatic cancer. Engineered exosomes, called iExosomes, are capable of carrying miRNA that targets and suppresses KRAS mutations, resulting in increased overall survival. These are more efficient at transporting cargo than liposomes because they evade immune attacks [133]. The Mitogen-activated protein kinase kinase kinase 9 (MAP3K9) is implicated in numerous biological processes and diseases, including cancer. Research has demonstrated that overexpression of miRNA-7 directly targets MAP3K9, resulting in the suppression of the MEK/ERK and NF-κB pathways [134] (Table 4 and Figure 3).

### 3.9. Immune Checkpoint Blockade Therapy in PDAC

The immune system plays a vital role in the fight against cancer. However, tumor cells have developed mechanisms to evade immune recognition, primarily through the immunosuppressive characteristics of the tumor microenvironment [121]. Immunotherapy, specifically targeting immune checkpoint molecules like programmed cell death protein-1 (PD-1), programmed death-ligand 1 (PD-L1) inhibitors, and cytotoxic T lymphocyte-associated antigen-4 (CTLA-4), has revolutionized cancer treatment by enhancing the immune response against tumors. Despite this progress, the effectiveness of current treatments remains limited [121]. CTLA-4, the initially discovered immune checkpoint molecule, possesses the ability to bind to B-7 ligands with high affinity. Consequently, CTLA-4 competes with the CD28 receptor for binding to the B-7 ligand, thereby disrupting the critical signaling required for T-cell activation. This competition results in an immunosuppressive effect, impairing the immune system’s ability to effectively respond to threats [122]. Ipilimumab, a humanized monoclonal IgG1 antibody, specifically targets CTLA-4 on T cells. By binding to CTLA-4 and blocking its interaction with ligands, Ipilimumab prevents the inhibitory signal that CTLA-4 typically sends to T cells. This blockade enhances T-cell activation and proliferation, ultimately promoting an immune response against tumors mediated by cytotoxic T-lymphocytes (CTLs) [123]. Similarly, tremelimumab (CP-675, CP-675,206) is also a humanized monoclonal IgG2 immunoglobulin antibody developed to target CTLA-4 molecules on T cells. By binding to CTLA-4, tremelimumab inhibits the immune checkpoint that mediates T-cell suppression. This inhibition promotes a cytotoxic T-lymphocyte antitumor immune response. Together, these therapies demonstrate the potential of overcoming immune checkpoint inhibition to effectively boost the body’s immune response against tumor cells [123].

The PD-1 immune checkpoint molecule is found on different types of immune cells and regulates T-cell function, similar to CTLA-4. It interacts with programmed death ligands PD-L1 and PD-L2 on antigen-presenting cells (APCs) and solid tumor cells. The strength of this interaction depends on the T-cell receptor (TCR) signal strength. When PD-1 binds to its ligands, it inhibits effector T-cell function by inhibiting kinases, reducing TCR signaling, and decreasing cytokine secretion such as INF-γ and IL-2 through phosphatase SHP1 and SHP2 phosphorylation [118,124,125]. Nivolumab is designed to specifically target PD-1 checkpoint molecules in immune cells like T cells, NK cells, and B cells. By interrupting the interactions between PD-1 and its ligands such as PD-L1 or PD-L2, it triggers the immune system, resulting in enhanced immune responses against tumors. Its capacity to enhance immune function represents a promising avenue in cancer therapy [123]. Pembrolizumab was approved by the FDA in 2014 as a PD-1/PD-L1 checkpoint inhibitor [127]. It has demonstrated efficacy in various cancers, such as renal cell carcinoma, gastrointestinal carcinomas, lung cancer, urothelial carcinoma, and metastatic melanoma [127]. The NCCN guidelines of 2021 further highlighted its importance, recommending it as a first-line therapy for patients with low-performance status, particularly those with MSI-H or dMMR metastatic pancreatic tumors. Additionally, it was suggested as a follow-up treatment for individuals with MSI-H or dMMR metastatic or locally advanced pancreatic cancers [128]. Durvalumab (MEDI4736) is an IgG1 antibody specifically designed to target the PD-L1 checkpoint ligand, which is present on solid tumors and tumor-infiltrating lymphocytes (TILs), for example, macrophages and dendritic cells. By binding to PD-L1, Durvalumab inhibits its interaction with PD-1, potentially activating T cells and enhancing the immune response against tumors expressing PD-L1 through the action of cytotoxic T lymphocytes (CTLs). Additionally, the Fc region of Durvalumab is modified to prevent antibody-dependent cytotoxicity and complement-dependent cytotoxicity [123,129]. BMS-936559, a humanized monoclonal antibody developed by Bristol-Myers Squibb targeting PD-L1, shows varying levels of efficacy in treating different types of advanced cancers [130].

## 4. Exosome-Based Delivery Systems of miRNA

EVs are diverse membrane-bound structures crucial for facilitating the transportation of biomolecules, including proteins, miRNA, and others, through the bloodstream [137]. On the surface of EVs, markers such as CD47 provide protection against phagocytic clearance. Additionally, modifying the surface of EVs enables the precise delivery of biomolecules to particular tissues. These unique characteristics make them suitable for conditional miRNA delivery [138].

Exosomes originate predominantly from late endosomes, giving them high efficacy in miRNA delivery [139]. Due to lower cytotoxicity and antigenicity, exosome-based delivery is highly effective [140]. Two approaches for loading miRNAs into exosomes are utilized. Firstly, modifying cell lines to overexpress the desired miRNA results in higher levels of miRNA secretion and subsequent encapsulation into exosomes. Alternatively, exosomes are isolated from cells and then enriched with specific miRNAs, typically achieved through transfecting adipose tissue-derived stem cells or mesenchymal stem cells with the target miRNA, leading to the production of exosomes containing the desired miRNA [141]. miRNA-enriched exosomes are utilized in a variety of diseases, including muscular disorders, brain disorders, cardiac disorders, and cancer. miRNAs, small interfering RNAs (siRNAs), and hairpin RNAs (shRNAs) are widely recognized as major therapeutic payloads that are commonly encapsulated in exosomes [142]. Researchers are currently engaged in the development of engineered exosomes, which carry therapeutic substances. A study by Zhou et al. showed that isolated EVs successfully loaded with paclitaxel and gemcitabine monophosphate resulted in exosomes exhibiting remarkable delivery capabilities and provided excellent targeted chemotherapeutic efficacy [143]. Similarly, a different set of exosomes was engineered to carry siRNA or shRNA molecules to specifically target KRAS mutation in pancreatic cancer. Following the implementation of this approach in mouse models, a significant reduction in tumor growth was observed as well as increased overall survival rates [133].

## 5. Conclusions

Pancreatic cancer is a big global health challenge that causes high mortality among cancer patients. Addressing the complexities of its diagnosis and treatment is imperative. EVs in the tumor microenvironment offer promising prospects for advancing diagnostic, prognostic, and therapeutic approaches in managing PDAC. Additionally, EVs encompass a diverse range of lipid-bound structures containing bioactive molecules, crucial for intercellular communication and maintaining cellular homeostasis. Exosomes carrying miRNAs play multifaceted roles in PDAC progression, influencing angiogenesis, migration, invasion, and metastasis. They modulate key pathways that have roles in PDAC, such as EMT, the Wnt pathway, and JAK/STAT, resulting in heightening cancer cell aggressiveness. Understanding these mechanisms could unveil potential therapeutic targets for combating pancreatic cancer metastasis and improving patient outcomes. Exosomal miRNA profiles offer promising avenues for precise diagnosis and prognosis. In PDAC, various miRNAs play an important role in conferring resistance to effective drugs such as gemcitabine, Cisplatin, doxorubicin, and 5-fluorouracil. They achieve this by altering various cellular processes such as apoptosis and drug metabolism, thereby facilitating this resistance. Understanding these mechanisms is critical for developing effective therapeutic strategies to overcome drug resistance and improve outcomes for pancreatic cancer patients. Furthermore, the potential of EVs as therapeutic agents and delivery systems has been demonstrated in various studies, underscoring their specificity and efficacy. However, further research and clinical trials are necessary before their widespread implementation in managing pancreatic cancer. 

## Figures and Tables

**Table 1:** Role of Exosome-miRNAs in Migration, invasion, and metastasis in PDAC.

miRNA	Expression Status	Source	Role of miRNA	Reference
miRNA-222	Up-regulation	PDAC cells	Promote PDAC cell invasion.	[42]
miRNA-501-3p	Overexpression	PDAC cells	promote PC cell invasion, migration, and metastasis.	[43]
miRNA-5703	Overexpression	Pancreatic stellate cells (PSCs)	Increase cell proliferation and migration.	[44]
miRNA-10a-5p	Overexpression	AsPC-1 and T3M4 cells	Enhance migration and invasion abilities.	[31]
miRNA-21	Overexpression	PDAC cells	Increase proliferation.	[32]
miRNA-125b-5p	Overexpression	Pancreatic cancer cells (PC-1.0)	Increase invasion and metastasis.	[45]
miRNA-301a	Overexpression	PDAC cells	Increase cell proliferation and metastasis.	[34]
miRNA-222	Overexpression	Pancreatic cancer cells	Promote invasion and metastasis.	[46]
miRNA-501-3p	Overexpression	TAMs cell	Enhance tumorigenesis and metastasis.	[36]

**Table 2:** Roles of Exosome-miRNAs in PDAC as Diagnosis biomarker.

S.No.	Cell Line/Number of Patients	Up-Regulated miRNA	Down-Regulated miRNA	Role of miRNA	Sources	Reference
1.	Patients samples 29	miR-10b, miR-21, miR-30c, and miR-181a	miR-let7a	Differentiate PDAC from Healthy.		[72]
2.	Only cell lines such as MiaPaca-2, Panc-1, and BxPC3	miR-21, miR-155, miR-221	miR-126	Differentiate between (PDAC) and normal pancreatic ductal epithelial cells.	Cell line	[75]
3.	Cell lines such as MIA PaCa-2, ANC-1, YAPC, and BxPC-3/Patients samples 15	miR-27a-3p, miR-221-3p, miR-23b-3p	miR-155-5p, let-7a-5p, miR-193a-3p	Predict poor prognosis of PDAC.	Serum/Plasma	[76]
4.		miR-30a-3p, miR-105	-	Predict better prognosis in PDAC patients.	PDAC specimens	[65]
5.	Patients samples 20	miR-106a, miR-18a	-	Significantly discriminated in PDAC patients and healthy.	PDAC plasma	[77]
6.	Patients samples 90	miR-142-5p and miR-204		Associated with better overall survival in pancreatic cancer.	Pancreatic tissue	[78]
7.	Patients samples 225	miR-212 and miR-675	miR-148a, miR-187, and Let-7g	Predict overall survival.	Pancreatic tissue	[66]
8.		miR-181b and miR-210		Discriminated PCa from normal individuals.	stool sample	[79]
9.	Cell line such as SW-1990, Panc-1, Miapaca-2 and Bxpc-3/Patients samples 21	miR-221/222	-	Differentiate between pancreatic ductal adenocarcinoma (PDAC) tissues and adjacent normal pancreatic tissues, as well as between invasive and nonaggressive pancreatic cancer cell lines.	Pancreatic tissue and cell line	[80]
10.	Only cell lines such as Aspc-1, BxPC-3	miR-301a-3p		Worse survival.	PDAC specimens and cell line	[81]
11.	Only Patients samples 69	21-5p, miR-485-3p, miR-708-5p, and miR-375		Distinguished PDAC from IPMN with a sensitivity and specificity of 95% and 85%, respectively.	FFPE pancreatic specimens	[82]
12.	Only Patients samples 90	miR-429		Significantly discriminated in PDAC patients and healthy.	PDAC plasma	[83]
13.	Only Patients samples 56	miR-451a		Prediction of recurrence and prognosis in PDAC patients.	PDAC plasma	[64]
14.	Only Patients samples 457	miR-486-5p and miR-938		Differentiate PDAC from healthy and chronic pancreatitis.	Plasma samples	[84]
15.	Only Patients samples 131	miR-1246, miR-4644, miR-3976, miR-4306		Differentiate PDAC from healthy.	serum-exosomes and exosome-depleted serum	[71]

**Table 3:** Exosome-miRNAs in overcoming drug resistance.

MiRNA	Patients/Cell Line	Expression	Function	Reference
miRNA-663a	Panc-1, BxPC-3, T3-M4, MIApaca-2	Overexpression	Overcome drug resistance to gemcitabine	[111]
miRNA-200b,miRNA200c and let-7	MiaPaCa-2, Panc-1, and Aspc-1	Up-regulation	Increase sensitivity to gemcitabine	[115]
miR-183	H6C7	Down-regulation	Increase sensitivity to 5-fluorouracil and gemcitabine	[116]
miR-3656, miR-509-5p, and miR-1243	No.of patients 157/FFPE(Pancreatic tissue)	Up-regulation	Increase sensitivity to gemcitabine	[114]
miRNA-181b	PDAC SW1990 and CFPAC-1	Up-regulation	Overcome drug resistance to gemcitabine	[117]
miRNA-181c	124 FFPE(Pancreatic tissue)/PANC-1 and BXPC3	Overexpression	Increase resistance to paclitaxel	[118]
miRNA-1291	PANC-1 xenograft and three different PC patient-derived xenograft (PDX) tumor mouse models		Increases sensitivity to gemcitabine-nab-paclitaxel combination therapy	[119]
miR-221 and miR-210	21/PANC-1, MIA PaCa-2, BXCP-3	Overexpression	Increase resistance to Lapatinib	[120]

**Table 4:** Exosome-miRNAs in PDAC treatment.

miRNA	Patients/Cell Line	Expression	Target	Function	Reference
miR-3607-3p	40/Mia PaCa-2 and PANC-1	Overexpression	Interleukin-26 (IL-26) in PC	Inhibit proliferation, migration, and invasion	[125]
miRNA-1231	BxPC-3 and MIA PaCa-2	Overexpression		Suppress aggressiveness of pancreatic cancer cells	[30]
miRNA-145	AsPC-1		OCT4, SOX2, NANOG, and KLF4	Decrease expression ITGA11, MAGEA4, SET, RPA1, MCM2, ABCC1, SPTBN1, and SPTLC. Inhibit pancreas carcinogenesis	[135]
miRNA-155	RInk-1	Knock down	EGFR, MT1-MMP, and K-ras	Reduce pancreatic cancer	[127]
miRNA-506	113	Up-regulation		Reduce disease progression	[129]
miRNA-142	42/PANC-1, SW1990, Hup, CFPAC-1 and a normal cell line HPC-Y5	Overexpression	HIF-1α	Inhibit and consequently reduce invasion and proliferation	[130]
miRNA-519d-3p	AsPC-1, BxPC3, PANC-1,SW1990, and HPNE	Up-regulation	RPS15A	Inhibit proliferation	[136]
miRNA-124		Overexpression	Rac1	Inhibit cell proliferation	[132]
miRNA-7	BxPC-3, PANC-1, and Patu-8988 cells, and HEK293T	Overexpression	MAP3K9	Inhibit cell proliferation	[134]
